# Early cardia neoplasms in the upper gastrointestinal tract have a higher detection rate under sedation: a retrospective cohort study based on the Chinese population

**DOI:** 10.3389/fmed.2026.1775066

**Published:** 2026-08-11

**Authors:** Xuanran Chen, Shunhai Zhou, Mingzhi Feng, Chaoyi Shi, Diyun Shen, Yan Sun, Yuzhen Bi, Jun Zhang

**Affiliations:** 1 The Second School of Clinical Medicine, Hangzhou Normal University, Hangzhou, Zhejiang, China; 2 The Second School of Clinical Medicine, Zhejiang Chinese Medical University, Hangzhou, Zhejiang, China; 3 Department of Gastroenterology, Center for General Practice Medicine, Zhejiang Provincial People’s Hospital (Affiliated People’s Hospital, Hangzhou Medical College), Hangzhou, Zhejiang, China; 4 The First School of Clinical Medicine, Zhejiang Chinese Medical University, Hangzhou, Zhejiang, China; 5 Department of Gastroenterology, Zhejiang Provincial People’s Hospital (Affiliated People’s Hospital, Hangzhou Medical College), Hangzhou, Zhejiang, China; 6 Department of Gastroenterology, Chun’an County First People’s Hospital (Zhejiang Provincial People’s Hospital, Chun’an Branch), Hangzhou, Zhejiang, China; 7 The First Affiliated Hospital of Zhejiang Chinese Medical University (Zhejiang Provincial Hospital of Traditional Chinese Medicine), Hangzhou, Zhejiang, China

**Keywords:** early adenocarcinoma of the gastric cardia, esophagogastroduodenoscopy, sedation, upper digestive tract early cancer, upper gastrointestinal precancerous lesions

## Abstract

**Background:**

Esophagogastroduodenoscopy (EGD) is a standard method for upper gastrointestinal (UGI) tumor screening. However, differing opinions persist regarding the impact of sedation on the detection rate of early adenocarcinoma of the gastric cardia (EGCA). This study evaluated the effect of sedation on the detection of EGCA, other upper digestive tract early cancer (UDTEC) and microscopic upper gastrointestinal (UGI) lesion detection.

**Methods:**

We retrospectively analyzed 313,503 symptomatic and screening patients undergoing EGD (2018–2025) at Zhejiang Provincial Hospital of Traditional Chinese Medicine and Zhejiang Provincial People’s Hospital. Histopathological biopsy data defined positive outcomes as EGCA and other UGI early cancers (esophageal, gastric, duodenal). Propensity score matching (PSM) assessed sedation-tumor detection associations. Primary outcomes included UGI precancerous lesions, UDTEC rates, and microscopic tumor yield (lesions/subjects). Secondary outcomes identified detection determinants via logistic regression.

**Results:**

Sedation was associated with modest but statistically significant higher detection rates of EGCA, overall UDTEC and UGI microscopic tumor foci compared with non-sedated EGD (EGCA: *p*< 0.001; UDTEC: *p*< 0.001; UGI microscopic tumor lesions: *p*< 0.001). Univariate/multivariate analyses identified gender, age, title of examining physician, years of practice, and examination time as independent detection predictors. After PSM (198,846 patients in total), sedation remained significantly associated with increased detection of EGCA (OR = 1.400, 95% CI 1.030–1.901, *p* = 0.026), overall UDTEC (OR = 1.196, 95% CI 1.102–1.295, *p*< 0.001) and microscopic tumor foci (OR = 6.861, 95% CI 3.278–14.367, *p* < 0.001).

**Conclusion:**

In this large retrospective cohort study of over 300,000 EGD subjects, sedation was associated with statistically significant but modestly higher detection rates of early gastric cardia adenocarcinoma, upper gastrointestinal precancerous lesions and overall upper digestive tract early cancers. The most prominent benefit of sedation was the improved detection of easily missed microscopic tumor foci. These findings support the consideration of sedation as a quality improvement option to enhance diagnostic yield for subtle early neoplasms, while acknowledging the relatively limited absolute gains in overall detection rates for unselected populations.

## Introduction

Upper gastrointestinal tract cancers (UGTC) impose a substantial global healthcare burden, particularly in East Asia (1, 2). Globally, the age-standardized incidence and mortality of gastric cancer are declining (3). However, due to population aging in East Asia (4), the absolute number of new cases and deaths continues to rise, creating a growing clinical burden. Therapeutic approaches and prognostic outcomes for UGTC are predominantly stage-dependent: early stage tumors are amenable to curative resection with favorable survival rates, whereas advanced-stage disease correlates with significantly poorer prognosis (5, 6). This critical importance of early diagnosis, with timely detection representing a pivotal window for intervention.

Upper gastrointestinal endoscopy (esophagogas- troduodenoscopy, EGD) is the gold standard for evaluating upper gastrointestinal (UGI) symptoms (7). Advances in endoscopic technology and widespread EGD adoption have enabled precise diagnosis of diverse clinical presentations. In Korea and Japan, EGD-based screening programs demonstrate improved survival rates through early gastric cancer detection (8, 9). Nevertheless, studies consistently report missed diagnoses and interval-stage progression in gastric/oesophageal cancers (10–12), with EGD false-negative rates estimated at 10–20% (13–16). These limitations underscore the imperative for rigorous quality assurance protocols in endoscopic screening practices.

The inherent invasiveness of EGD induces physiological stress responses that may compromise procedural accuracy and diagnostic precision (17). In contrast, painless EGD demonstrates clinical advantages through enhanced hemodynamic stability and reduced procedural stress (18). Although sedation improves patient compliance and tolerability (19, 20). The impact of sedation on upper digestive tract early cancer (UDTEC) detection rates and the detection rates of different endoscopic lesions in the upper gastrointestinal tract remains controversial. Recent clinical observations suggest potential diagnostic benefits of sedation in detecting superficial UGTC (21), while research studies indicate that sedation negatively impacts the assessment of the esophagogastric junction during esophagogastroduodenoscopy (22), there is currently no conclusive evidence to analyze these two differing viewpoints. No studies have systematically evaluated the effect of sedation protocols on UDTEC detection rates, particularly for early adenocarcinoma of the gastric cardia (EGCA). This critical knowledge gap challenges the presumed correlation between sedation protocols and endoscopic diagnostic efficacy.

The primary aim of this study was to systematically evaluate the independent effect of sedation on the detection rates of EGCA, UGI precancerous lesions and overall UDTEC, with a specific focus on microscopic tumor foci—the most easily missed lesions in routine endoscopy. The primary endpoints included the detection rates of the above lesions, compared between groups using propensity score matching (PSM). The secondary endpoint was to identify independent predictors of lesion detection via logistic regression. This study aims to provide evidence for clinical decision-making of sedation protocols, rather than directly recommending universal routine sedation.

## Materials and methods

### Study design and participants

Endoscopic and histopathological data from patients undergoing upper gastrointestinal endoscopy between June 2018 and May 2025 were retrospectively collected from the endoscopy centers of the First Affiliated Hospital of Zhejiang University of Chinese Medicine and Zhejiang Provincial People’s Hospital.

Inclusion criteria encompassed: (1) presentation of upper gastrointestinal symptoms (epigastric discomfort, bloating, pain, heartburn, acid reflux, dysphagia, choking, hiccups, unexplained appetite loss, weight reduction, or anemia); (2) high-risk population screening; (3) age = 18 years; or (4) routine health evaluations. Exclusion criteria comprised: (1) Patients with a definitive diagnosis and undergoing precision endoscopy or endoscopic treatment; (2) history of surgical management for upper gastrointestinal malignancies; (3) contraindications for gastroscopy tolerance; (4) patients with severe underlying diseases who have no independent choice between sedated and non-sedated Esophagogastroduodenoscopy (EGD) (only eligible for a single procedure type); or (5) EGD procedures without concurrent biopsy sampling.

### Pre-inspection preparation

Prior to the gastroscopy, patients are required to fast for 6–8 h to ensure that there is no residual food in the stomach and to facilitate a clear view of the gastric mucosa. In addition, patients are required to discontinue anticoagulant medications (e.g., warfarin, aspirin, etc.) for 5–7 days, adjusted according to the doctor’s instructions, to minimize the risk of bleeding. Thirty minutes before the examination, the patient may receive local anesthesia, usually with lidocaine spray or gel applied to the throat to reduce discomfort during insertion. Pronase (Manufactured by Beijing Tide Pharmaceutical Co., Ltd., National Drug Approval No. H20110030, Specification: 20,000 units/sachet) at a dose of 20,000 units was orally administered 15 min before the examination after being stirred and dissolved, to enhance gastric mucosal visibility. The examinees were performed by endoscopists from each center. Patients in the non-sedation group underwent gastroscopy in the left lateral decubitus position after pharyngeal numbness. All patients receiving sedation shall undergo intravenous anesthesia based on a combination of remifentanil 0.3 μg/kg and propofol 2.0 mg/kg, administered by a dedicated team of sedation physicians without endotracheal intubation. Patients in the sedation group received propofol-based sedation and underwent EGD when they lost consciousness, had no relevant reflexes, were muscle-relaxed, and had stable vital signs. During the examination, low-flow oxygen was maintained via a nasal cannula.

### Basic data collection

Data extracted from electronic medical records included: patient demographics (age, gender), procedural parameters (examination timeframe, endoscopic equipment generation), operator characteristics (endoscopist professional rank, clinical experience duration), histopathological outcomes, anesthesia protocols, and concomitant findings (e.g., neoplastic lesions). Ethical approval was obtained from the Ethics Review Boards (Protocol No.: 2025-KSL-043-01).

Analytical variables were structured as follows: procedural timing (morning: 00:00–12:00; afternoon: 12:01–23:59); scheduling context (workdays vs. non-workdays). Endoscopist variables encompassed professional hierarchy (Resident, Attending, Associate Chief, and Chief Physicians) and clinical experience tiers (<5, 5–10, >10 years).

### Endoscopic standards

All endoscopic procedures and diagnostic evaluations were performed by board-certified endoscopists at both participating centers. All EGDs were performed using GIF-H260 and GIF-H290 endoscopies (Olympus Optical Co., Ltd., Tokyo, Japan). Standardized protocols mandated systematic photographic documentation of key anatomical landmarks including the pharynx, esophagus, cardia, gastric fundus, corpus, angulus, antrum, greater/lesser curvatures, and duodenal segments (anterior/posterior walls and descending portion). After detecting a lesion during a standard Olympus gastroscopic examination, the endoscopist switched to a magnifying endoscope for narrow-band imaging (NBI). The entire procedure was performed by the same experienced endoscopist. Following insertion, the lesion was systematically observed under both white light and magnifying endoscopy with NBI (NBI-ME) to evaluate its extent, gross morphology, microvascular patterns, and pit patterns. When early upper gastrointestinal cancer or precancerous lesions were suspected, targeted biopsies were performed on the most prominent areas of the lesion under NBI-ME guidance. Histopathological confirmation served as the diagnostic gold standard for upper digestive tract early cancer (UDTEC) identification.

### Detection criteria

Precancerous lesions were defined as histopathological alterations demonstrating direct association with upper gastrointestinal (UGI) neoplasia, encompassing intraepithelial neoplasia (IN). IN was stratified into low-grade intraepithelial neoplasia (LGIN) and high-grade intraepithelial neoplasia (HGIN). Systematic biopsies were obtained for endoscopically suspected neoplastic lesions during screening EGD. When HGIN was histologically identified without definitive mucosal/submucosal layer differentiation, endoscopic submucosal dissection (ESD) specimens were required for confirmatory diagnosis (23). Early esophageal cancers (EEC) were strictly defined as malignancies confined to the mucosal layer via ESD histopathology (23). Early gastric cancers (EGC) included lesions limited to mucosal or submucosal layers (24), while gastric cardia adenocarcinoma (GCA), defined as cancer involving the esophagogastric junction with epicenter within 2 cm into the proximal stomach (25). The current operational definition for UGI microscopic tumor foci remains unclear in clinical practice. We categorized tumor size into the following groups for analysis and evaluation: <1 cm, 1–2 cm, and >2 cm. All histopathological assessments were conducted through dual independent reviews by senior pathologists using resection specimens or biopsy samples.

### Statistics

This retrospective cohort study was conducted on endoscopic and histopathological data obtained from patients who received upper gastrointestinal endoscopy examinations at the First Affiliated Hospital of Zhejiang University of Traditional Chinese Medicine and Zhejiang Provincial People’s Hospital (June 2018 to May 2025). Categorical measures were summarized using frequency distributions with percentage values. Data exhibiting normal distribution underwent independent *t*-tests and were reported as mean ± standard deviation. Count data were analyzed through R×C contingency chi-square tests, presented as counts with corresponding percentages. Fisher’s exact test was applied when the expected frequency in any cell of the contingency table was < 5. Continuous variables were compared between groups via independent *t*-tests. Categorical variables underwent intergroup analysis using chi-square tests.

Propensity score matching addressed confounding variables through 1:1 nearest-neighbor pairing (caliper width: 0.001 logit propensity score), achieving balance across age, gender, endoscopist experience duration, and procedural timing. Comparisons within matched pairs employed paired chi-square tests and Wilcoxon signed-rank tests. Statistical adjustments maintained covariate equilibrium between comparison groups during analytical procedures. For the propensity score-matched cohort, conditional logistic regression was used to estimateodds ratios (OR) and 95% confidence intervals (95% CI), accounting for the paired study design.

Binary logistic regression modeling identified factors influencing early adenocarcinoma of the gastric cardia (EGCA), UGI precancerous lesion and other UDTEC detection rates, with outcomes presented through adjusted OR and 95% CI. The relationship between sedation and the screening rates of EGCA, UGI precancerous lesions and other UDTEC was assessed using the entry method by correcting for all variables in binary logistic regression analyses.

For all analyses reporting OR and 95% CI, the no-sedation group was defined as the reference category.

All analyses were performed using SPSS 27.0 software for data analysis.

## Results

### Patient characteristics

**FIGURE 1:**
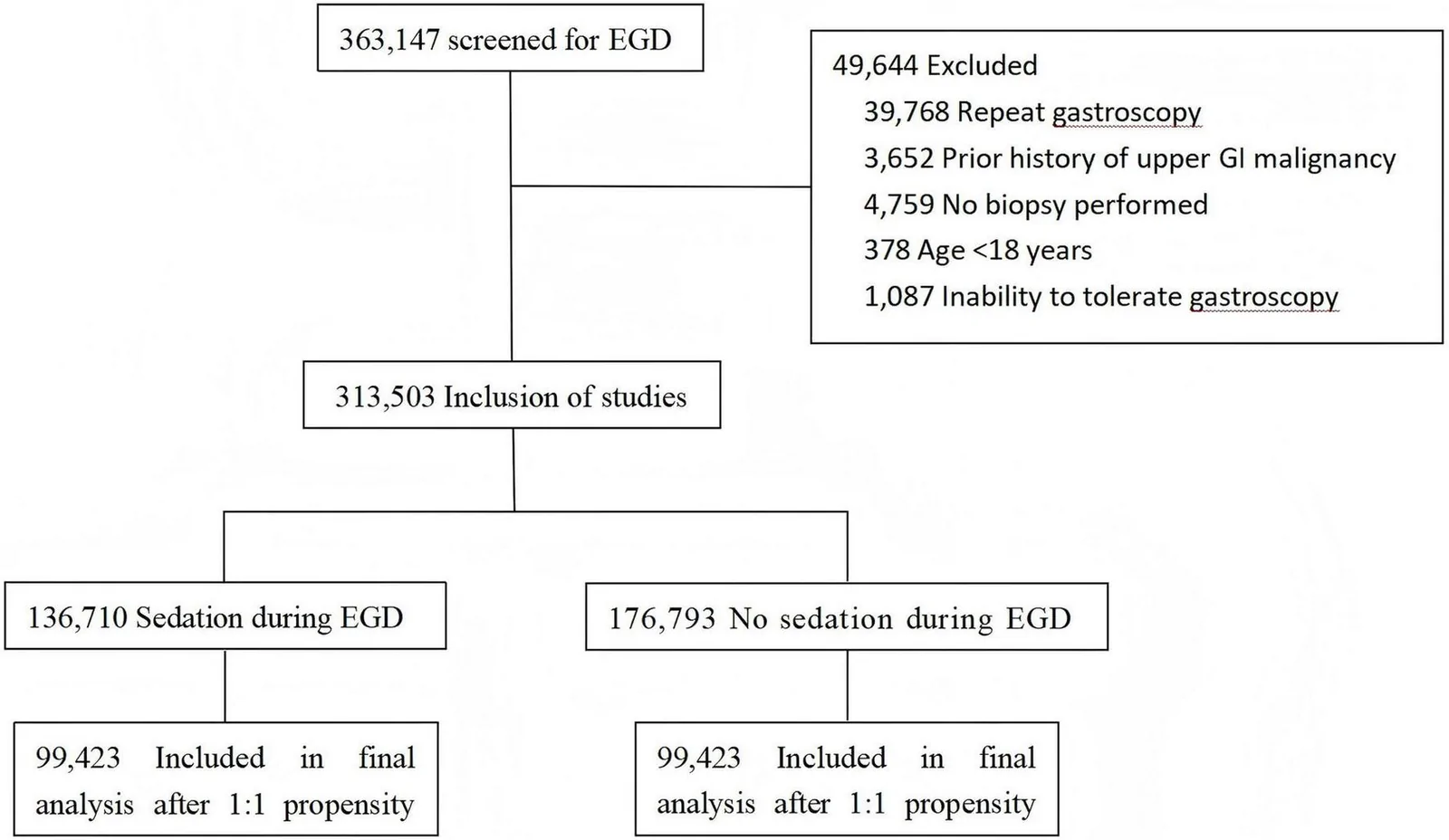
Flow diagram of the study population. *This retrospective cohort study included patients undergoing diagnostic EGD from two medical centers between June 2018 and May 2025. Eligible participants were allocated to the sedation group or no-sedation group according to actual clinical practice. Patients were excluded stepwise per prespecified criteria, and 1:1 PSM was performed to balance baseline covariates between the two groups, yielding the final analytical cohort.

The multicenter database initially contained 363,147 patients undergoing EGD. Application of exclusion criteria eliminated 49,644 cases (Figure 1). The final analytical cohort comprised 313,503 patients with a mean age of 50.25 years. Gender distribution showed 144,936 male and 168,567 female participants. The no-sedation group contained 136,710 individuals (mean age 48.93 ± 15.63 years; 66,543 male, 70,167 female). The sedation group included 176,793 participants (mean age 51.28 ± 13.69 years; 78,393 male, 98,400 female). Compared with the non-sedation group, the sedation group had an older mean age, a higher proportion of female patients, a higher proportion of procedures performed by attending and chief physicians, longer clinical experience of endoscopists, and a higher proportion of afternoon examinations, with statistically significant differences in the above baseline characteristics between the two groups (*p*< 0.05). Detailed baseline data are shown in Table 1.

**TABLE 1:** Baseline characteristics and endoscopic findings in the normal and sedated groups.

Group	Variable	No-sedation	Sedation
Number		136,710	176,793
Sex	Male	66,543 (48.76%)	78,393 (44.34%)
	Female	70,167 (51.36%)	98,400 (55.66%)
Age, yr	Average	48.93±15.63	51.28±13.69
	<40	42,006 (30.73%)	38,238 (21.63%)
	40–65	73,314 (53.63%)	110,508 (62.51%)
	>65	21,192 (15.50%)	27,600 (15.61%)
Title of endoscopists	Resident physician	26,751 (19.57%)	18,195 (10.30%)
	Attending physician	24,810 (18.15%)	49,941 (28.26%)
	Associate chief physician	40,707 (29.79%)	47,979 (27.16%)
	Chief physician	44,442 (32.52%)	60,597 (34.29%)
Length of time in practice	<5 Year	27,102 (19.83%)	18,840 (10.66%)
	5–10 Year	37,122 (27.16%)	62,403 (35.32%)
	>10 Year	72,486 (53.04%)	95,469 (54.02%)
Examination time	Morning	129,600 (94.83%)	98,865 (55.94%)
	Afternoon	7,110 (5.20%)	77,847 (44.06%)
	Weekday	124,095 (90.80%)	176,304 (99.77%)
	Nonwork days	12,615 (9.23%)	408 (0.02%)

### Clinical results

The screening rate of early adenocarcinoma of the gastric cardia (EGCA) in the sedation group was significantly higher than that of the no-sedation group (OR = 1.553, 95% CI 1.205–1.996, *p*< 0.001). The screening rate of LGIN and HGIN in the sedation group was significantly higher than that of the no-sedation group (LGIN: OR = 1.653, 95% CI 1.547–1.767, *p*< 0.001; HGIN: OR = 1.438, 95% CI 1.313–1.574, *p*<0.001). The screening rate of EGC and EEC in the sedation group was significantly higher than that of the no-sedation group (EGC: OR = 1.479, 95% CI 1.371–1.591, *p*< 0.001; EEC: OR = 1.173, 95% CI 0.999–1.378, *p* = 0.035); but there was no significant difference between the screening rate of the sedation group in early duodenal cancer (EDC) and that of the no-sedation group (OR = 1.221, 95% CI 0.884–1.684, *p* = 0.195); and the screening rate of the painless sedation group was significantly higher than that of the no-sedation group in overall early cancer (OR = 1.414, 95% CI 1.324–1.511, *p*< 0.001). For the different sites of EGC, the screening rate in the sedation group was significantly higher than that in the normal group in the fundus (OR = 1.745, 95% CI 1.213–2.506, *p* = 0.002), body (OR = 1.298, 95% CI 1.095–1.539, *p* = 0.002), angle (OR = 1.498, 95% CI 1.235–1.817, *p*< 0.001) and sinus (OR = 1.498, 95% CI 1.354–1.657, *p*< 0.001) (Table 2).

**TABLE 2:** Relationship between sedation and lesion detection rate and UDTEC screening rate.

Group	Variable	No-sedation	Sedation	*t/*X^2^	*P*-value	OR	95% CI
Lesion types	Low-grade intraepithelial neoplasia	1,278 (0.94%)	2,751 (1.55%)	234.527	<0.001	1.653	1.547–1.767
	High-grade intraepithelial neoplasia	720 (0.52%)	1,353 (0.75%)	66.839	<0.001	1.438	1.313–1.574
	Early esophageal cancer	246 (0.18%)	378 (0.21%)	4.451	0.035	1.173	0.999–1.378
	Early gastric cancer	1,068 (0.78%)	2,061 (1.17%)	115.38	<0.001	1.479	1.371–1.591
	Early stage duodenal cancer	60 (0.04%)	96 (0.05%)	1.681	0.195	1.221	0.884–1.684
Location distribution of EGC	EGCA	90 (0.07%)	183 (0.10%)	12.579	<0.001	1.553	1.205–1.996
	Fundus	42 (0.03%)	96 (0.05%)	9.741	0.002	1.745	1.213–2.506
	Body	210 (0.15%)	357 (0.20%)	9.971	0.002	1.298	1.095–1.539
	Antrum	156 (0.11%)	306 (0.17%)	18.221	<0.001	1.498	1.235–1.817
	Sinus	570 (0.42%)	1,116 (0.63%)	66.194	<0.001	1.498	1.354–1.657
	Total	1,374 (1.00%)	2,535 (1.43%)	115.140	<0.001	1.414	1.324–1.511

OR, odds ratio; CI, confidence interval; EGC, early gastric cancer; EGCA, early adenocarcinoma of the gastric cardia. **All ORs and 95% CIs were calculated with the no-sedation group as the reference category.

We included the above factors in a one-way logistic regression analysis and multifactorial logistic regression analysis showing that gender, age, title of endoscopists, years of practice, and examination time were independent risk factors for the detection rate of early cancer in the upper gastrointestinal tract in the general and sedation groups (Table 3, and detailed results in Supplementary Table 1).

**TABLE 3:** Univariate and multifactorial logistic regression analysis of UDTEC detection rate in normal and sedated groups.

Group	Univariate logistic regression			Multifactorial logistic regression		
	*P*	OR	95% CI	*P*	OR	95% CI
Sex	<0.001	1.19	1.173–1.207	<0.001	1.236	1.217–1.257
Age, yr	<0.001	1.011	1.011–1.012	<0.001	1.003	1.003–1.004
Title of endoscopists	<0.001	1.096	1.089–1.104	<0.001	0.821	0.806–0.836
Length of time in practice	<0.001	1.211	1.199–1.222	<0.001	1.428	1.391–1.466
Date of examination	<0.001	0.023	0.021–0.025	<0.001	0.036	0.032–0.039
Testing time	<0.0001	14.373	14.009–14.747	<0.001	13.337	12.995–13.688

OR, odds ratio; CI, confidence interval; PSM, propensity score matching. **All ORs were calculated with the no-sedation group as the reference category.

### Propensity matching results

Based on possible confounding variables, a total of 198,690 (99,345 pairs) participants were propensity score-matched (1:1) after a propensity score matching (PSM) with a caliper of 0.001. Of the matched participants, the mean age was 50.29 years; 89,690 (44.16%) were male and 108,900 (55.84%) were female. Matching showed no significant difference in gender, age, title of examining physician, years of practice, and duration of examination in the general and sedation groups. Table 4 shows the baseline characteristics of participants after propensity score matching.

**TABLE 4:** Patient characteristics of the propensity-matched cohort.

Group	Variable	No-sedation	Sedation	*t*/X^2^	*P*-value
Number		99,345	99,345		
Sex	Male	44,845 (45.14%)	44,850 (45.14%)	0.000	0.982
	Female	54,500 (54.86%)	54,495 (54.86%)		
Age, yr	Average	50.29±14.28	50.29±14.28		1.000
Title of endoscopists	Resident physician	11,837 (11.92%)	11,781 (11.86%)	0.216	0.972
	Attending physician	21,875 (22.02%)	21,894 (22.04%)		
	Associate chief physician	30,147 (30.34%)	30,210 (30.41%)		
	Chief physician	35,486 (35.72%)	35,460 (35.69%)		
Length of time in practice	<5 Year	12,119 (12.20%)	12,063 (12.14%)	0.232	0.891
	5–10 Year	29,234 (29.42%)	29,310 (29.5%)		
	>10 Year	57,992 (58.37%)	57,972 (58.35%)		
Examination time	Morning	92,247 (92.86%)	92,247 (92.86%)	0.000 0.000	1.000 1.000
	Afternoon	7,098 (7.14%)	7,098 (7.14%)		
	Weekday	99,033 (99.69%)	99,033 (99.69%)		
	Nonworkdays	291 (0.31%)	291 (0.31%)		

OR, odds ratio; CI, confidence interval. **All ORs were calculated with the no-sedation group as the reference category.

After propensity score matching, the detection rate of EGCA was 0.07% in the no-sedation group and 0.10% in the sedation group, and sedation was associated with higher odds of EGCA detection (OR = 1.400, 95% CI 1.030–1.901, *p* = 0.026). For precancerous lesions, the detection rates of LGIN were 0.99% in the no-sedation group and 1.45% in the sedation group (OR = 1.465, 95% CI 1.351–1.589, *p*< 0.001), and the detection rates of HGIN were 0.59 and 0.73%, respectively (OR = 1.239, 95% CI 1.111–1.381, *p*< 0.001). And the screening rate of the painless sedation group was significantly higher than that of the no-sedation group in overall early cancer (1.10% vs. 1.32%, OR = 1.196, 95% CI 1.102–1.295, *p*< 0.001). The detection rate of EGC was 0.84% in the no-sedation group and 1.09% in the sedation group (OR = 1.289, 95% CI 1.178–1.411, *p*< 0.001); no significant difference was observed in EEC detection between the two groups (0.20% vs. 0.19%, OR = 0.911, 95% CI 0.746–1.112, *p* = 0.360), nor in EDC detection (0.05% vs. 0.04%, OR = 0.815, 95% CI 0.547–1.213, *p* = 0.312).

**FIGURE 2:**
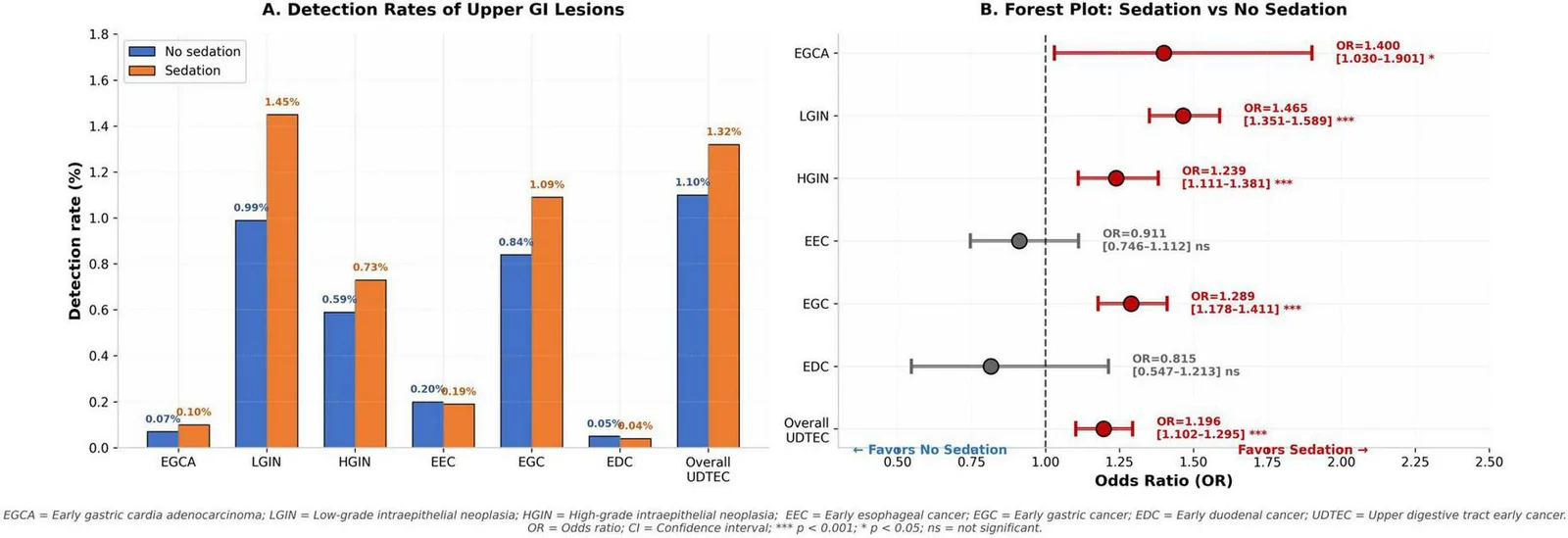
Detection rates of upper GI lesions after propensity score matching. **(A)** Detection rates of upper GI lesions. **(B)** Forest plot: sedation vs. no sedation. * In this figure shows the absolute detection rates (%) of different upper gastrointestinal lesions in the sedation group and no-sedation group. Panel B presents a forest plot of OR with 95% CI for lesion detection, with the no-sedation group set as the reference category. Statistical significance: ***p<0.001; *p<0.05; ns, not statistically significant. ** EGCA, early gastric cardia adenocarcinoma; LGIN, low-grade intraepithelial neoplasia; HGIN, high-grade intraepithelial neoplasia; EEC, early esophageal cancer; EGC, early gastric cancer; EDC, early duodenal cancer; UDTEC, upper digestive tract early cancer.

For different anatomical sites of EGC, no significant differences were found in the fundus (0.03% vs. 0.05%, OR = 1.297, 95% CI 0.845–1.991, *p* = 0.233) and body (0.16% vs. 0.19%, OR = 1.174, 95% CI 0.951–1.448, *p* = 0.134) between groups; while sedation was associated with higher detection rates in the angle (0.12% vs. 0.18%, OR = 1.557, 95% CI 1.231–1.966, *p*< 0.001) and sinus (0.46% vs. 0.57%, OR = 1.244, 95% CI 1.100–1.408, *p*< 0.001) (Table 5 and Figure 2).

**TABLE 5:** Relationship between sedation and lesion detection rate and UDTEC screening rate after propensity matching.

Group	Variable	No-sedation	Sedation	*t*/X^2^	*P*-value	OR	95% CI
Lesion types	Low-grade intraepithelial neoplasia	982 (0.99%)	1,439 (1.45%)	87.330	<0.001	1.465	1.351–1.589
	High-grade intraepithelial neoplasia	589 (0.59%)	730 (0.73%)	15.171	<0.001	1.239	1.111–1.381
	Early esophageal cancer	203 (0.20%)	185 (0.19%)	0.837	0.360	0.911	0.746–1.112
	Early gastric cancer	837 (0.84%)	1,079 (1.09%)	31.584	<0.001	1.289	1.178–1.411
	Early stage duodenal cancer	54 (0.05%)	44 (0.04%)	1.021	0.312	0.815	0.547–1.213
Location distribution of EGC	EGCA	70 (0.07%)	98 (0.10%)	4.981	0.026	1.400	1.030–1.901
	Fundus	37 (0.03%)	48 (0.05%)	1.424	0.233	1.297	0.845–1.991
	Body	161 (0.16%)	189 (0.19%)	2.244	0.134	1.174	0.951–1.448
	Antrum	115 (0.12%)	179 (0.18%)	14.738	<0.001	1.557	1.231–1.966
	Sinus	454 (0.46%)	565 (0.57%)	12.154	<0.001	1.244	1.100–1.408
	Total	1,094 (1.10%)	1,308 (1.32%)	19.820	<0.001	1.196	1.102–1.295

OR, odds ratio; CI, confidence interval; EGC, early gastric cancer; EGCA, early adenocarcinoma of the gastric cardia. **All ORs were calculated with the no-sedation group as the reference category.

### Comparison of the screening rate of microscopic tumor foci in upper gastrointestinal (UGI) between the normal group and the sedation group

**FIGURE 3:**
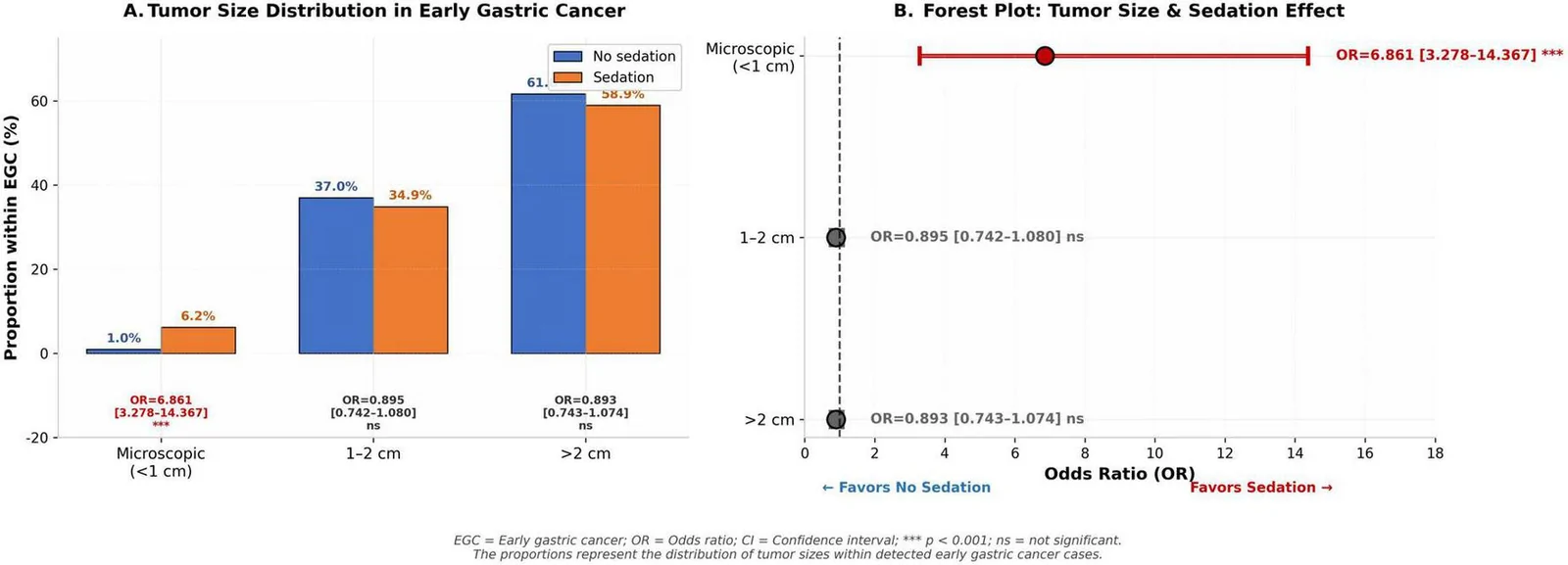
Comparison of the screening rate of microscopic tumor Foci in the upper GI tract between the sedation and no-sedation groups. **(A)** Tumor size distribution in early gastric cancer. **(B)** Forest plot: tumor size and sedation effect. * In this figure shows the size distribution of lesions among all pathologically confirmed EGC cases, presented as the proportion (%) of each size subgroup within detected EGC cases. Panel B presents a forest plot of OR with 95% CI for the detection of lesions of different sizes, with the no-sedation group set as the reference category. Statistical significance: ***p<0.001; ns, not statistically significant. EGC, early gastric cancer; OR, odds ratio; CI, confidence interval.

After propensity matching, the screening rate of early gastric cancer in the no-sedation group was 837 cases (0.84%); among them, there were 8 cases (0.96%) of microscopic tumor foci (OR = 6.861, 95% CI 3.278–14.367, *p*< 0.001); 313 cases (36.95%) of 1–2 cm tumor foci (OR = 0.895, 95% CI 0.742–1.080, *p* = 0.249); and 516 cases (61.65%) of >2 cm tumor foci (OR = 0.893, 95% CI 0.743–1.074, *p* = 0.221). The screening rate of early gastric cancer in the sedation group was 1,079 cases (1.09%); among them, 67 cases (6.21%) were microscopic tumor foci; 376 cases (34.85%) of 1–2 cm tumor foci; and 636 cases (58.94%) of >2 cm tumor foci (Table 6 and Figure 3).

**TABLE 6:** Comparison of the screening rate of microscopic tumor Foci in the upper GI tract between the sedation and no-sedation groups.

Group	Variable	No-sedation	Sedation	*t*/X^2^	*P*-value	OR	95% CI
After PSM	Early gastric cancer	837	1,079	34.430	<0.001	6.861	3.278–14.367
	Microscopic tumor foci	8 (0.96%)	67(6.21%)				
	1–2 cm Tumor foci	313 (36.95%)	376 (34.85%)	1.326	0.249	0.895	0.742–1.080
	>2 cm tumor foci.	516 (61.65%)	636 (58.94%)	1.495	0.221	0.893	0.743–1.074

OR, odds ratio; CI, confidence interval; PSM, propensity score matching. **All ORs and 95% CIs were calculated with the no-sedation group as the reference category. ***Data before propensity score matching are presented in Supplementary Table 2.

## Discussion

We demonstrated a significant association between sedation administration during EGD procedures and enhanced detection rates of early adenocarcinoma of the gastric cardia (EGCA), upper gastrointestinal (UGI) precancerous lesion and other upper digestive tract early cancer (UDTEC). Comparative analysis showed higher diagnostic yield in the sedated cohort versus the non-sedated group, though the absolute difference was modest. Propensity score-matched analysis corroborated these findings, confirming statistically higher detection rates for EGCA, UGI precancerous lesion and other UDTEC in the sedation group. However, as clearly shown in the propensity score matching (PSM) analysis, the absolute increases in overall detection rates were relatively modest, with sedation associated with a 40% higher odds of EGCA detection (OR = 1.400, 95% CI 1.030–1.901) and a 19.6% higher odds of overall UDTEC detection (OR = 1.196, 95% CI 1.102–1.295). Despite the small absolute differences, the findings carry notable clinical and public health relevance. For EGCA in particular—which is often asymptomatic at early stage and has a dismal prognosis once progressing to advanced stage—an absolute increase of 0.03% translates to 3 additional early stage cases detected per 10,000 screening individuals. In large-scale population-based screening programs, this would prevent a considerable number of interval cancers and cancer-related deaths, justifying the clinical value of the statistically significant improvement. Our results suggest that sedation may serve as a quality improvement measure to further elevate the diagnostic yield of population-based endoscopic screening programs.

Disease stage directly determines UGTC prognosis and treatment efficacy, highlighting the critical need for early detection. EGD is the global primary tool for upper gastrointestinal precancerous lesions and early esophageal squamous cell carcinoma (ESCC) detection, with diagnostic accuracy as a core quality indicator (23). Gastroscopy’s inherent invasiveness often induces physiological stress, causing patient reluctance and delayed diagnosis (26, 27). Optimized sedation reduces discomfort, improves compliance and mucosal visualization to boost diagnostic precision (20, 28, 29), enabling pain-free, rapidly anxiolytic exams with better safety and tolerability (30–32). The 2019 Asian Consensus confirms sedation enhances superficial esophagogastric tumor detection (21), yet 75% of European EGDs are still performed without sedation despite its global use (33). Emerging evidence confirms pharmacological interventions boost EGD diagnostic yield: multicenter trials show isoproterenol significantly increases superficial tumor detection (88.89% vs. 33.33%, *p* = 0.048) (34); multiple studies confirm sedation enhances detection of esophageal squamous cell carcinoma (ESCC), precancerous lesions and small polyps (35–37), with Wu et al. (35) attributing this to longer exam time, standardized techniques and systematic biopsy.

However, sedation’s effect on gastroesophageal junction (EGJ) visualization remains controversial. Kim et al. (22) found midazolam or propofol sedation impairs EGJ inspection, prolonging procedures and worsening Z-line visualization. The EGJ is supported by two sphincters: the smooth muscle lower esophageal sphincter (LES) and skeletal crural diaphragm (38). Its inspiratory pinchcock contraction is key to optimal Z-line visualization (39), but propofol and midazolam’s sedative properties may suppress this mechanism, impairing EGJ view (40). Multiple studies confirm these agents reduce LES pressure, promote reflux, and inhibit diaphragmatic contractility (41, 42). The discrepancy between these prior negative findings and our positive results for EGCA detection can be explained by three key differences in study design and practice protocols: (1) The improved patient cooperation under sedation allows for more stable and thorough application of Narrow Band Imaging (NBI), enabling clearer visualization of mucosal microvascular and pit patterns, which enhances the detection rate of early cancers (43). (2) Although visualization of the cardia and dentate line remains suboptimal under sedation, sedation eliminates patient discomfort and gag reflexes, ensuring sufficient withdrawal time for a comprehensive and meticulous examination of the cardia without interruption, thereby reducing the risk of missed diagnoses due to a hurried procedure. (3) Endoscopists from both centers have undergone rigorous standardized training and are highly experienced, demonstrating a heightened awareness of early cardiac cancer and adhering to more standardized and meticulous procedures.

Liang et al. (44) found no significant improvement in early gastric cancer detection with sedation, and Dominitz et al. (45) reported comparable precancerous lesion/early ESCC detection rates regardless of sedation status, with adjusted models showing no sedation-lesion detection association after covariate control. Our findings diverge, showing optimized sedation significantly improves UDTEC and precancerous lesion detection, though PSM analysis found no significant early esophageal cancer detection difference between groups (*p*> 0.05). The reasons for this may include: (1) The straight anatomy of the esophageal lumen allows for relatively clear observation even without sedation. (2) Both centers involved in this study employed NBI for meticulous examination of the esophageal lumen during endoscope withdrawal, which facilitated the detection of esophageal lesions. Given the limited number of events in some subgroups (e.g., early duodenal cancer), we did not perform an interaction test between sedation and tumor site to avoid unreliable results due to insufficient statistical power; the site-stratified results presented in this study can preliminarily reflect the differential benefits of sedation across anatomical sites.

Subcentimeter (<1 cm) lesions are highly susceptible to missed detection in standard EGD, making improved microscopic lesion detection critical for optimizing endoscopic quality and early tumor identification. Existing evidence confirms sedation-enhanced EGD has superior diagnostic performance for small lesions: Chang et al. (36) reported a threefold higher UGI microtumor detection rate (0.16% vs. 0.05%, *p* = 0.023) in a 20,052-patient EGD cohort; a 10,940-patient retrospective study also found sedation improved 5-mm diminutive polyp detection (15.8% vs. 5.6%, *p*< 0.001) (46). Our study yielded consistent results, showing sedation markedly enhances microscopic tumor detection versus the non-sedated group, with our far larger cohort further strengthening the credibility of these findings.

For safety, current evidence confirms good tolerability of propofol-based sedation in diagnostic EGD, with an extremely low incidence of transient cardiovascular and respiratory adverse events (47–50). Paradoxically, some studies link EGD sedation to lower cardiovascular/cerebrovascular (CCV) event rates: EGD scope insertion induces physiological stress via upper airway compression and oxygen desaturation (51, 52), provoking adverse cardiorespiratory changes (53, 54), yet sedation mitigates these responses without raising baseline risks. Though diagnostic endoscopy sedation is an independent CCV risk factor (OR = 1.12, 95% CI 1.09–1.14, *p*< 0.001) (55), its clinical impact is negligible across adult cohorts; Ross and Newton (56) found sustained late-procedure blood pressure elevation in elderly EGD patients via continuous noninvasive monitoring. Advanced age (>65 years) significantly increases CCV event risks post-EGD compared to younger patients (40–65 years) (57), so comorbidities, age and underlying health must be considered to evaluate gastroscopy feasibility and modality. These findings support optimized sedation to improve procedural tolerance while preserving cardiovascular safety. In clinical practice, individualized evaluation of sedation indications is recommended. For young and low-risk patients, sedation can be preferentially recommended to improve the detection rate of subtle lesions; for elderly patients and those with severe underlying cardio-cerebrovascular diseases, the benefits and risks of sedation should be carefully weighed, and personalized sedation protocols should be formulated. Future studies can further explore stratified sedation strategies based on population characteristics to optimize clinical decision-making (56, 57). In our study, age emerged as an independent risk factor affecting the screening rate for UDTEC through standard/painless gastroscopy.

Structured endoscopic training programs have been shown to significantly improve early gastric cancer detection rates (58), with multicenter evidence confirming enhanced diagnostic yield following standardized educational interventions (59). Paradoxically, operator experience levels demonstrate no significant correlation with baseline detection rates of UGI precancerous lesions and early cancers (EC) (60). Our analysis identified both clinical experience duration (<5 vs. 5–10 vs. >10 years) and professional hierarchy (Resident vs. Attending vs. Associate/Chief) as independent predictors influencing UGI precancerous lesion and EC detection efficacy. These findings collectively suggest that targeted competency-based training, rather than experiential seniority alone, may optimize endoscopic screening performance.

Histological upgrading from LGIN to HGIN or EC frequently occurs following endoscopic resection (ESD/EMR) (61). Notably, LGIN carriers exhibit substantially elevated gastric cancer risks, demonstrating 25- and 8-fold increased malignancy probabilities compared to the general population and undifferentiated lesion cohorts, respectively (62, 63). These findings underscore the critical importance of precise LGIN detection for intercepting pathological progression and enabling early EC diagnosis. Our data revealed enhanced LGIN identification through sedation-assisted gastroscopy, facilitating implementation of intensified surveillance protocols to mitigate disease advancement.

This investigation acknowledges several methodological constraints: (1) The retrospective design inherently limits control over unmeasured confounding variables, including Helicobacter pylori infection status, atrophic gastritis grade, history of smoking and alcohol consumption, family history of upper gastrointestinal cancer, total endoscopic withdrawal time, and endoscopy indication, which are proven to be associated with early upper GI cancer detection and may confound the observed association. (2) Exclusive use of propofol-based sedation may introduce selection bias, though existing evidence suggests minimal association between sedation types and post-procedural cardiovascular complications (57). (3) Geographical generalizability is restricted by sole inclusion of Chinese participants, necessitating validation in populations with divergent UDTEC epidemiology. Sedation benefits may demonstrate context-dependent variability across regions with differing UGI cancer burdens. (4) Absence of sedation-related adverse event documentation precludes comprehensive safety profiling–a critical component of procedural risk-benefit analysis. (5) Lack of longitudinal follow-up impedes comparative assessment of interval cancer rates between cohorts. (6) This study only included EGD cases with concurrent biopsy sampling, as cases without biopsy lack histopathological diagnosis for core endpoints; the number of non-biopsy cases was extremely small (approximately 370 cases), and inclusion would introduce significant sample heterogeneity, which may affect the robustness of the results. (7) This study was unable to present the overall TNM staging and T1a/T1b stratification of all included early cancer cases, as complete TNM staging requires systematic follow-up and imaging data, and accurate T stratification depends on postoperative pathological results of endoscopic resection, which are not available for biopsy-only diagnosed cases. (8) This study lacks complete long-term follow-up data of the full cohort, so the association between sedation and post-endoscopy upper gastrointestinal interval cancer cannot be analyzed. (9) The historical recording of endoscopy indication lacks unified standardized specifications, so it cannot be included in the regression model for confounding adjustment; the ultra-large sample size has mitigated the impact of unsystematic bias to a certain extent. (10) This study did not include health economic indicators such as procedure cost, anesthesiologist staffing input and detailed procedure duration, and thus cannot conduct cost-effectiveness analysis of sedated gastroscopy.

Taken together, sedation was associated with statistically significant but modestly higher odds of detecting EGCA, UGI precancerous lesions and overall UDTEC, and a substantially enhanced ability to identify easily missed microscopic tumor foci during EGD.

## Data Availability

The original contributions presented in this study are included in the article/Supplementary material, further inquiries can be directed to the corresponding author.
