# Supplementary material for: Early cardia neoplasms in the upper gastrointestinal tract have a higher detection rate under sedation: a retrospective cohort study based on the Chinese population
Source: Front Med (Lausanne). 2026 Aug 11;13:1775066. doi: 10.3389/fmed.2026.1775066 (PMC13503191; doi:10.3389/fmed.2026.1775066)
Supplement: Supplementary file 1 [file Supplementary_file_1.doc]

**Supplementary Table 1** Univariate and Multifactorial logistic regression analysis of UDTEC detection rate in normal and sedated groups

|  |  | **B** | **SE** | **Wald X^2^** | **P** | **OR** | **95%CI** |
| --- | --- | --- | --- | --- | --- | --- | --- |
| Univariate  logistic  regression | Sex | 0.174 | 0.007 | 581.862 | <0.001 | 1.19 | 1.173-1.207 |
|  | Age, yr | 0.011 | 0.0001 | 1984.813 | <0.001 | 1.011 | 1.011-1.012 |
|  | Title of endoscopists | 0.092 | 0.003 | 723.319 | <0.001 | 1.096 | 1.089-1.104 |
|  | Length of time in practice | 0.191 | 0.005 | 1493.214 | <0.001 | 1.211 | 1.199-1.222 |
|  | Date of examination | -3.783 | 0.050 | 5625.464 | <0.001 | 0.023 | 0.021-0.025 |
|  | Testing time | 2.664 | 0.013 | 41431.428 | <0.0001 | 14.373 | 14.009-14.747 |
| Multifactorial logistic regression | Sex | 0.212 | 0.008 | 667.730 | <0.001 | 1.236 | 1.217-1.257 |
|  | Age, yr | 0.003 | 0.0001 | 132.303 | <0.001 | 1.003 | 1.003-1.004 |
|  | Title of endoscopists | -0.197 | 0.009 | 453.327 | <0.001 | 0.821 | 0.806-0.836 |
|  | Length of time in practice | 0.356 | 0.013 | 714.559 | <0.001 | 1.428 | 1.391-1.466 |
|  | Date of examination | -3.338 | 0.051 | 4204.620 | <0.001 | 0.036 | 0.032-0.039 |
|  | Testing time | 2.591 | 0.013 | 38208.095 | <0.0001 | 13.337 | 12.995-13.688 |

*Abbreviations: OR, odds ratio; CI, confidence interval; PSM, propensity score matching. **All ORs were calculated with the no-sedation group as the reference category.

**Supplementary Table 2** Microtumor detection rates in the normal and sedated groups before PSM

| **Group** |  | **No-sedation** | **Sedation** | t/X^2^ | P value | OR | 95%CI |
| --- | --- | --- | --- | --- | --- | --- | --- |
| Before PSM | Early gastric cancer | 1068 | 2061 |  |  |  |  |
|  | microscopic tumor foci | 9（0.84%） | 118（5.72%） | 42.970 | <0.001 | 7.146 | 3.615-14.138 |
|  | 1-2cm tumor foci | 418（39.14%） | 765（37.12%） | 1.185 | 0.276 | 0.918 | 0.789-1.069 |
|  | >2 cm tumor foci | 641（60.02%） | 1178（57.16%） | 2.336 | 0.126 | 0.889 | 0.765-1.034 |
